# Biomechanics and hydrodynamics of prey capture in the Chinese giant salamander reveal a high-performance jaw-powered suction feeding mechanism

**DOI:** 10.1098/rsif.2012.1028

**Published:** 2013-05-06

**Authors:** Egon Heiss, Nikolay Natchev, Michaela Gumpenberger, Anton Weissenbacher, Sam Van Wassenbergh

**Affiliations:** 1 Department of Biology, University of Antwerp, Antwerp 2610, Belgium; 2 Department of Integrative Zoology, University of Vienna, Vienna 1090, Austria; 3 Clinic of Diagnostic Imaging, University of Veterinary Medicine Vienna, Vienna 1210, Austria; 4 Zoo Vienna, Vienna 1130, Austria; 5 Evolutionary Morphology of Vertebrates, Ghent University, Ghent 9000, Belgium

**Keywords:** *Andrias davidianus*, suction feeding, computed tomography, high-speed video, computational fluid dynamics, Urodela

## Abstract

During the evolutionary transition from fish to tetrapods, a shift from uni- to bidirectional suction feeding systems followed a reduction in the gill apparatus. Such a shift can still be observed during metamorphosis of salamanders, although many adult salamanders retain their aquatic lifestyle and feed by high-performance suction. Unfortunately, little is known about the interplay between jaws and hyobranchial motions to generate bidirectional suction flows. Here, we study the cranial morphology, as well as kinematic and hydrodynamic aspects related to prey capture in the Chinese giant salamander (*Andrias davidianus*). Compared with fish and previously studied amphibians, *A. davidianus* uses an alternative suction mechanism that mainly relies on accelerating water by separating the ‘plates’ formed by the long and broad upper and lower jaw surfaces. Computational fluid dynamics simulations, based on three-dimensional morphology and kinematical data from high-speed videos, indicate that the viscerocranial elements mainly serve to accommodate the water that was given a sufficient anterior-to-posterior impulse beforehand by powerful jaw separation. We hypothesize that this modified way of generating suction is primitive for salamanders, and that this behaviour could have played an important role in the evolution of terrestrial life in vertebrates by releasing mechanical constraints on the hyobranchial system.

## 1. Introduction

A key component of aquatic prey capture in most vertebrates is an explosive expansion of the oropharyngeal cavity by a series of coordinated movements of head parts. Owing to the incompressibility of water, this expansion causes prey and surrounding water to be drawn into the open mouth [1], a behaviour referred to as suction feeding. The widespread use of suction feeding among aquatic vertebrates proves that it is a very effective way to capture a wide range of prey [2–10]. Most suction feeding fish species use a fast motion of the hyoid and abduction of the gill cover as the main contributor to suction generation by oropharyngeal expansion [1–3,11]. Such an expansion causes the prey and surrounding water to accelerate into the gaping mouth, and the engulfed water is then expelled through the gill slits [11]. Obviously, such a unidirectional suction flow is restricted to animals with gills and gill slits, such as fishes or larval amphibians [1–3,6,7,9,11–18].

Metamorphosed amphibians and other tetrapods with reduced gills and closed gill slits evolved a bidirectional suction flow system where the inflowing water is first stored in the expanded pharyngo-esophageal cavity and then slowly released through the slightly opened mouth again [6,8,10–18]. While many well-conceived studies have been conducted on unidirectional suction flow systems, both in fishes [1–3,19–22] and larval salamanders [12–18], our biomechanical knowledge on bidirectional systems, although used by a great variety of aquatic tetrapods [4–6,8,10,23–25], is lagging behind. The dynamics and water flow patterns of bidirectional suction feeding in salamanders, for example, have not been quantified yet. Nevertheless, the change from a unidirectional to a bidirectional feeding system is considered a key innovation behind the fish–tetrapod transition during terrestrial evolution of vertebrates.

Previous research suggested that the feeding system of one of the most primitive groups, the Cryptobranchidae or giant salamanders, is significantly distinct from other aquatic salamander groups [26]. This multi-variate comparative analysis of feeding kinematics and morphology placed *Cryptobranchus alleganiensis*, which is one of the three extant species of giant salamanders, separate from five more derived salamander groups. In *Cryptobranchus*, a considerable delay was observed in the onset of the depression of the hyoid [26], generally a key component for generating suction by expanding the oropharyngeal cavity [11,21]. However, the functional consequences of this altered feeding pattern remain unknown. This calls for a more detailed analysis to unravel the biomechanical roles of the oral jaws and hyobranchial elements during suction generation in giant salamanders.

Also from an evolutionary point of view, Cryptobranchidae are of particular interest. As these extant salamanders are considered one of the groups with the most ancestral characteristics [27–30] and with an origin dating back to over 161 Myr [30], they may allow us to gain insights into the ancestral aquatic feeding system of urodeles. Furthermore, since the early rise of amphibians is characterized by large, salamander-like aquatic predators with large, flat and broad skulls (similar to the extant giant salamanders), extant cryptobranchids are ideal candidates to infer function and behaviour of early amphibians [31,32].

The aim of this study was to elucidate how the largest giant salamander, the Chinese giant salamander *Andrias davidianus*, captures its aquatic prey. We will initially focus on the functional morphology of the jaws and hyobranchium, as these are considered essential elements in suction generation. Given the extensively ossified hyobranchium in other aquatic salamander taxa [6], we expect a well-developed and strongly mineralized hyobranchium also to occur in the permanently aquatic *A. davidianus*. We further predict that the large overall body size, along with the relatively large, broad and flat head characteristic for giant salamanders, will have a strong impact on the way suction is generated. More specifically, we want to find out whether the previously observed kinematic pattern of the jaw and hyoid of *Cryptobranchus alleganiensis* [26] is also present in *A. davidianus*, how this relates to the species' morphology, and what the functional implications are in terms of hydrodynamics. The purpose of the hydrodynamic analysis is to evaluate the impact of a separation of this species' long and broad jaws on the fluid mechanics of suction feeding.

## 2. Material and methods

The Chinese giant salamander, *A. davidianus*, is the largest living amphibian. It reaches a maximum length of over 160 cm and a weight of 50 kg, and lives in rivers and streams of central and eastern China where it feeds on elusive prey such as fish and crayfish [33]. *Andrias davidianus* develops from a gill-bearing larva that undergoes metamorphosis where gills are reduced and gill slits closed.

### 2.1. Computed tomography

To analyse the *in situ* three-dimensional morphology of skull and hyobranchial skeleton, a computed tomography (CT) scan was performed on a freeze-dried specimen (127 cm total length), kindly provided by the Zoological Collection of the Department of Theoretical Biology, University of Vienna (stock no. 1/2009). The specimen was scanned by a Somatom emotion multi-slice scanner (Siemens AG, Germany) using 130 kV, 100 mA and 0.6 mm thick axial slices. For three-dimensional reconstruction and visualization, the resulting greyscale image stacks were imported into Amira v. 4.1 software (Mercury Computer Systems, Chelmsford, MA, USA). Surfaces of the bony structures were created with the IsoSurface tool of Amira. The cartilaginous parts of the hyoid as well as muscles were surface reconstructed by labelling them manually, followed by the generation of a surface. Surface optimizations were performed by iterated steps of triangle reduction and smoothing. Snapshots of the reconstructions were taken with the Amira software.

### 2.2. High-speed video recordings and kinematics

**Figure 1.**
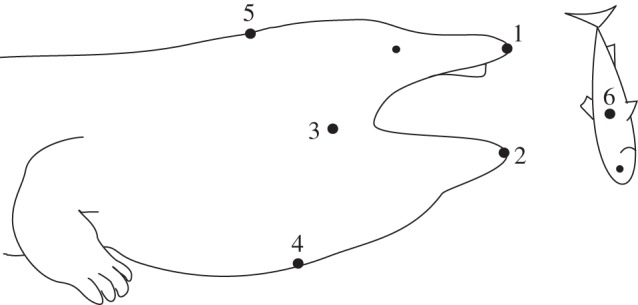
Landmarks used in the kinematic analysis: 1, upper jaw tip; 2, lower jaw tip; 3, jaw joint; 4, hyobranchium; 5, nape; 6, estimated centre of mass of prey.

Two postmetamorphic *A. davidianus* were provided by the Zoo Vienna (62 cm and 114 cm total length), and one (118 cm total length) by the Aqua Terra Zoo Vienna. The animals were filmed in their home aquaria in the zoo facilities in lateral view. To facilitate lateral filming, hiding structures (big plastic tubes or artificial rocks) were placed parallel to the front windows. Whole or halved dead roach (*Rutilus rutilus*), or trout pieces (*Oncorhynchus mykiss*) of comparable size (all prey items ranged from 8 to 14 cm length), suspended from a thin cotton thread, were offered to the giant salamanders approximately 40 cm in front of their hiding structures and 3–5 cm above ground. The suction strike was recorded with a Photron Fastcam-X 1024 PCI (Photron Limited, Tokyo, Japan) digital high-speed camera at 2000–6000 Hz with two dedocool spotlights (Dedo Weigert Film, GmbH, Munich, Germany) as the light source. From a total of 41 recordings, 24 (eight recordings for each individual) were chosen based on their strict lateral view for kinematical analysis. The horizontal (*x*-axis) and vertical (*y*-axis) coordinates of previously defined landmarks (shown in figure 1) were tracked frame by frame using SIMI-MatchiX software (SIMI Reality Motion Systems, Germany). Our landmarks were based on those used by other studies on salamander prey capture [15,34–38] to allow direct comparisons of kinematics. According to the two-dimensional displacements of the landmarks, we calculated the following movements: jaw movement (distance between the tips of the upper and the lower jaw from start of mouth opening until mouth closing); hyoid depression (distance between neck and throat where maximum depression occurs); movement of the prey towards the salamander mouth (change of *x*-value of prey landmark from the start of prey movement until it enters the mouth); angle displacements of skull (head elevation from start of mouth opening until maximum gape) and lower jaw (lower jaw depression from start of mouth opening until maximum gape) as well as mean and maximum velocities and accelerations of each movement.

In order to account for variability of the prey capture behaviour between the three individuals, and according to previous research on prey capture biomechanics in salamanders [15,34–36], we selected 11 variables obtained from the kinematic analysis that best described the whole prey capture event: maximum gape distance, maximum hyoid depression, duration mouth opening, duration mouth closing, duration gape cycle, time of hyoid depression start (equal to the delay of hyoid depression relative to start of mouth opening), duration hyoid depression, mean velocity of mouth opening, mean velocity of mouth closing, mean velocity of hyoid depression and mean velocity induced to the prey through suction. After calculating descriptive statistics for each variable and individual, we checked for normal distribution of their residuals and as they were normally distributed, we performed a multi-variate analysis (MANOVA). The individual was treated as the fixed factor and the variables as random factors. In order to account for repeated measurements, simultaneous Bonferroni correction was used to adjust significance levels to *p* ≤ 0.0046. Furthermore, we tested for a correlation between the timings of mouth opening and hyoid depression. All statistical analyses were performed on a PC with the SPSS Statistics v. 20 software package (IBM, USA).

In order to account for strike-to-strike variability and to avoid the potential confounding effects of kinematic means, the time axis of each prey capture sequence was scaled to the maximum gape (mouth opening) value in the kinematic graphs. Given that one of the three individuals was significantly smaller and its values differed significantly from both the larger individuals (as shown in the corresponding results part), it was not included in the kinematic profile shown in the results.

To calculate the amount of sucked water volume, the head of one salamander (114 cm total length), from snout tip to the pectoral girdle, was approximated by a series of elliptical cylinders. The lengths of the major and minor axes of the ellipses correspond to head height and width, respectively. The amount of sucked water volume was considered as the difference between head volume just before suction (before mouth opening started) and just after mouth closure when the buccal cavity was maximally expanded. No significant lateral expansion of the oropharynx was assumed.

### 2.3. Computational fluid dynamics

**Figure 2.**
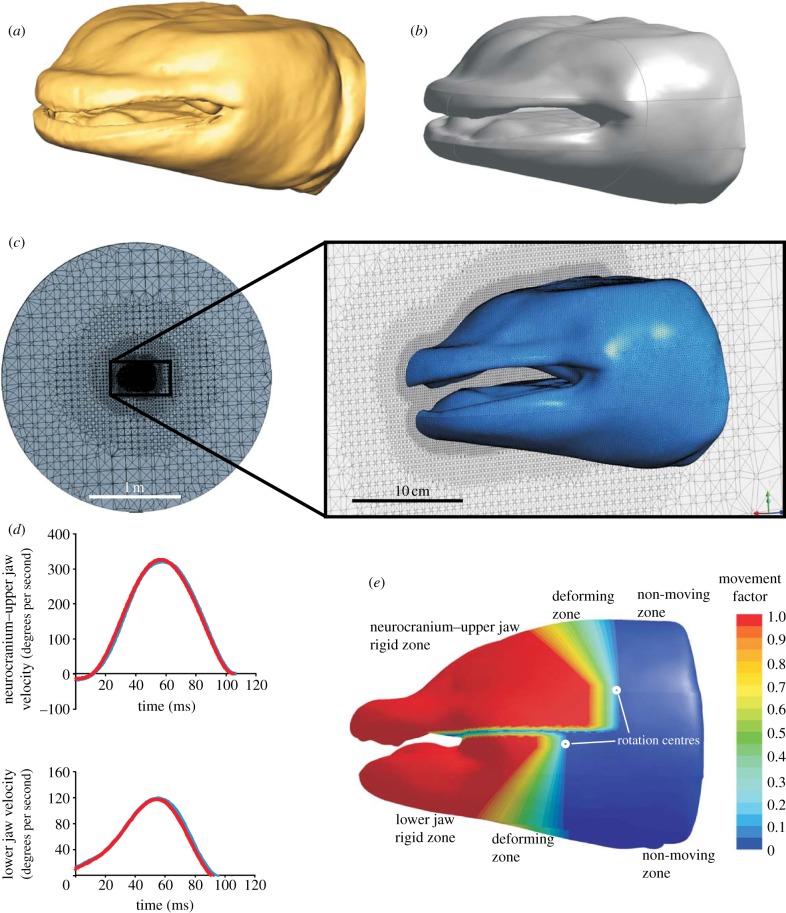
Different steps of CFD modelling. (*a*) The external and intra-oral surface of the head was generated by a CT scan of a freeze-dried Chinese giant salamander. Through reverse engineering software, this surface was segmented and fitted with a series of spline (NURBS) surfaces (*b*) onto which a surface grid was generated. (*c*) The salamander head model was centred in a spherical flow domain, an unstructured, tetrahedral mesh was created, boundary conditions were set (boundary sphere, pressure outlet; salamander surface, no-slip wall), and imported into the CFD solver. (*d*) Rotations of the neurocranium upper jaw and lower jaw were programmed as grid-deformation scripts that included polynomial fits (red curves) of the smoothed kinematic data (blue curves). (*e*) To allow smooth movement of the grid without grid intersections, transitional zones were defined where the grid deformed by assigning a motion factor ranging from 1 (completely following the rotation of neurocranium upper jaw or lower jaw) to 0 (grid nodes stay at their initial place).

For the computational fluid dynamics (CFD) simulation, we used a giant salamander head model based on the CT scan (outer and inner surfaces; figure 2*a*). These CT scan surfaces were first converted into a single watertight surface using Geomagic Qualify v. 10 software (Geomagic, NC, USA), and transformed into non-uniform rational B spline surfaces (separating functional units such as interior and exterior surfaces of the lower and upper jaws, and a narrow middle zone in between; figure 2*b*) using VRMesh Studio v. 5.0 (VirtualGrid, Seattle, WA, USA). Next, the salamander head model was centred in a spherical flow domain boundary with a radius of 1.5 m, and the space surrounding the head was meshed with 4 858 032 tetrahedral cells (size of 2.5 mm at the head surface and 0.25 m at the outer domain boundary and a growth rate of 1.2 between the two; figure 2*c*), using the patch independent algorithm in ANSYS Meshing v. 14.0 (ANSYS, Canonburg, PA, USA). The model was solved for unsteady, laminar flow in ANSYS Fluent v. 14.0 with a time step of 1 ms. As transition to turbulent flow is very unlikely to occur because of the short duration and high accelerations of the water during suction feeding [20], a laminar flow model was chosen. The motion and deformation of the mesh was included by a user-defined function. The simulated movements of the head were based on the data obtained from our high-speed videos (i.e. angular velocities of the neurocranium and lower jaw), of which the profiles were accurately fitted with sixth-order polynomial functions (figure 2*d*). For a smooth deformation of the grid generated around the head, values of polynomial fits were multiplied by a factor ranging from 1 to 0 (shown in figure 2*e*). Changes in the mesh surrounding the head over time were automatically performed by ANSYS Fluent, using spring-based smoothing and re-meshing algorithms. A grid-convergence test indicated that a further refinement of our mesh would have introduced a large computational cost, but would not change the results by more than 5 per cent. For example, refining from 1 to 2 million cells resulted in a change by 5 per cent in both the calculated peak velocity and peak pressure, whereas a further refinement from 2 to 4.5 million cells only resulted in a 1.7 per cent lower peak velocity and a 2.9 per cent lower peak sub-ambient pressure magnitude. During this final refinement, the timing of the peak negative value and the zero-crossing of pressure remained unchanged. Also, time-step size convergence was confirmed: smoothed replicas of the velocity and pressure profiles of a simulation using 2 ms as time-step size were calculated with the used time steps of 1 ms. The results were post-processed using ANSYS CFD-Post v. 14.0.

## 3. Results

### 3.1. Morphology

**Figure 3.**
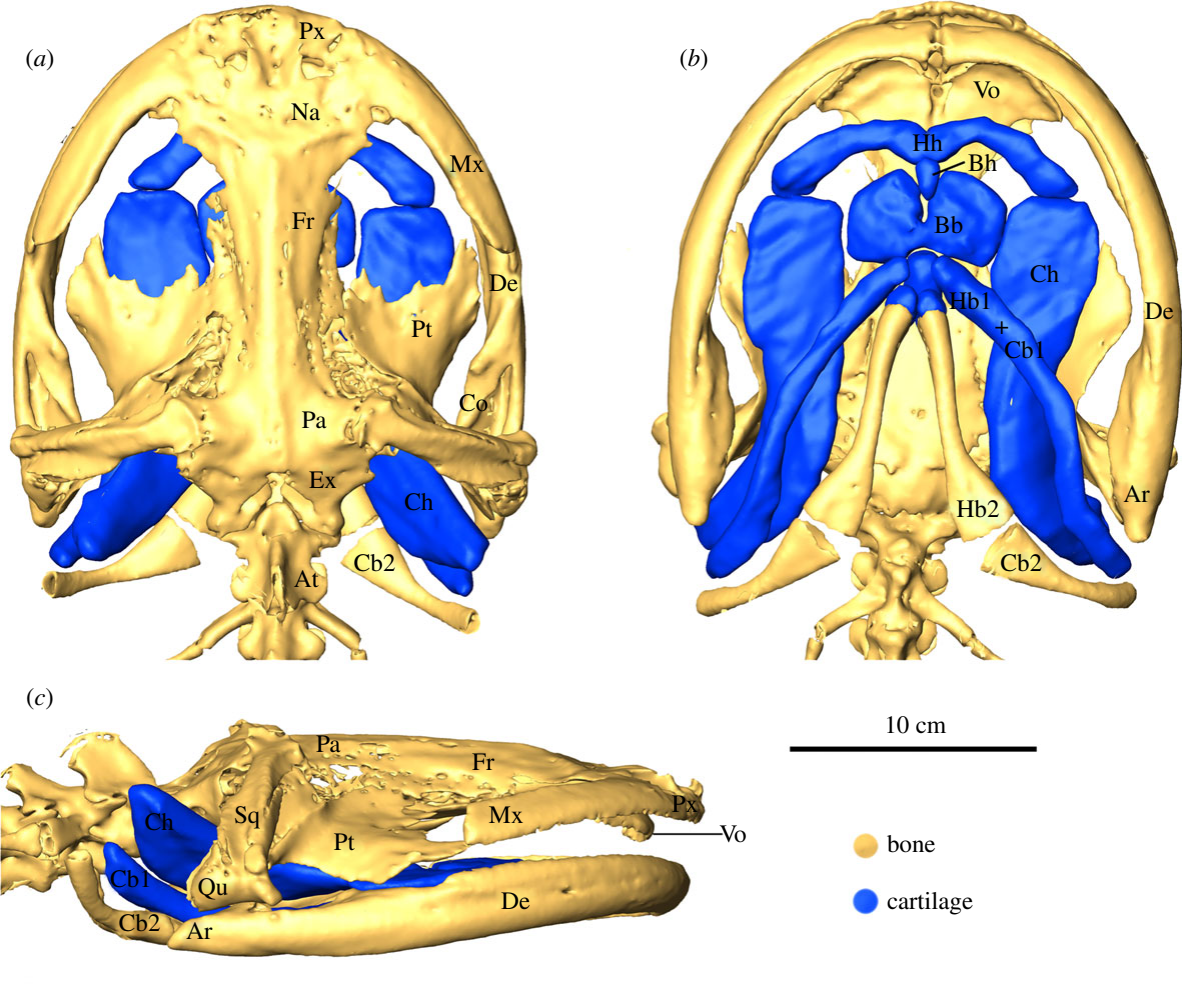
Three-dimensional reconstructions of the head skeleton of *Andrias davidianus* in (*a*) dorsal, (*b*) ventral and (*c*) lateral view. Note the broad and flat skull (Ar, articular; At, atlas; Co, coronoid; De, dentary; Ex, exoccipital; Fr, frontal; Mx, maxilla; Na, nasal; Pa, parietal; Pt, pteygoid; Px, premaxilla; Qu, quadrate; Sq, squamosal; Vo, vomer) and the mainly cartilaginous hyobranchium that covers the broad space between both dentaries (Bb, basibranchial; Bh, basihyal; Cb1, ceratobranchial 1; Cb2, ceratobranchial 2; Ch, ceratohyal; Hb1, hypobranchial 1; Hb2, hypobranchial 2; Hh, hypohyal).

The skull of *A. davidianus* is very broad and flat. The upper jaw consists of the premaxilla, maxilla and vomer (figure 3*a–c*). The premaxilla and the maxilla bear a continuous, nearly hemispherical row of teeth that is followed posteriorly by the second row of vomerine teeth. Dorsally, behind the premaxilla, lie the paired nasal, frontal and parietal bones that build up the roof of the braincase. The braincase is enclosed posteriorly by the exoccipitals, which bear the occipital condyles that articulate with the first vertebra, the atlas. The floor of the braincase is formed by the large parasphenoid that lies posterior to the vomer and runs posteriorly to the exoccipitals. Amphilateral to the parasphenoid lie both flattened pterygoids that build up the lateral roof of the oropharyngeal cavity. The pterygoids attach posteriorly to the squamosal and quadrate, and these three elements together assemble the suspensorium. The suspensorium articulates with the articular element of the mandible that is attached anteriorly to the tooth bearing dentary.

The hyobranchial apparatus in postmetamorphic *A. davidianus* lies in the oropharyngeal floor and broadly covers the space between both dentaries of the lower jaw (figure 3*b*). It is built up by the hyoid arch and the first two branchial arches, which remain mainly cartilaginous. Only elements of the second branchial arch are mineralized. The anterior-most elements of the hyobranchium, the hypohyals, show a bow-like shape and are medially fused together. Posterior to the medial fusion lies the small and (from the ventral view) oval basihyal. Posterior to the distal tips of the hypohyals lie the large flat ceratohyals that extent posteriorly beyond the jaw joint. Between the anterior portions of the ceratohyals, and posterior to the small basihyal lies the broad and remarkably flat basibranchial that is followed posteriorly by the elongated hypobranchial 1, fused with the ceratobranchial 1, and the only mineralized elements found in the hyobranchial skeleton of *A. davidianus*: hypobranchial 2 that articulates with a distinct ceratobranchial 2.

**Figure 4.**
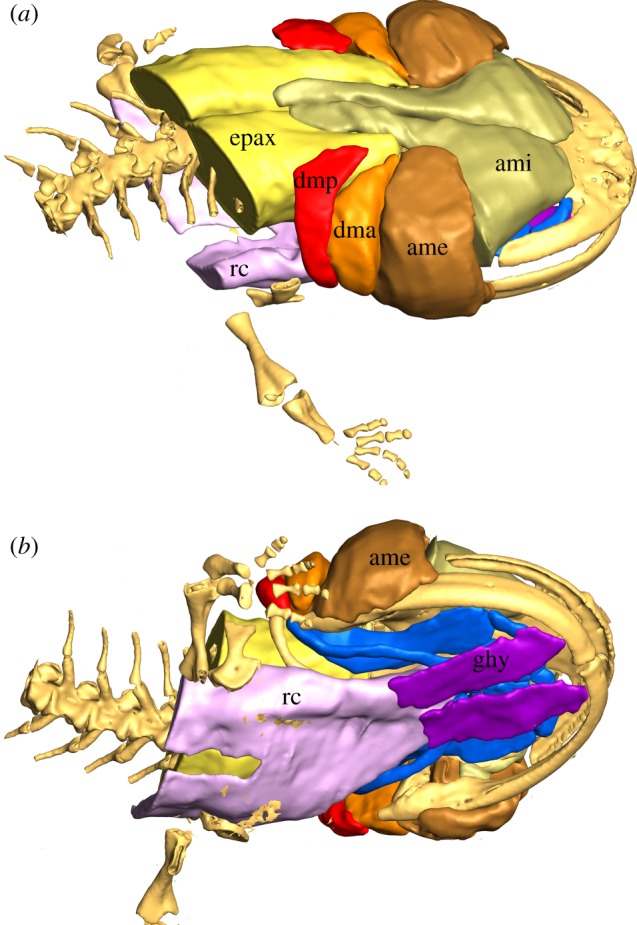
Three-dimensional volume reconstruction of the main muscles involved in prey capture in *A. davidianus* from (*a*) dorsolateral and (*b*) ventrolateral view: ame, M. adductor mandibulae externus; ami, M. adductor mandibulae internus; dma, M. depressor mandibulae anterior; dmp, M. depressor mandibulae posterior; epax, epaxialis musculature; ghy, M. geniohyoideus; rc, M. rectus cervicis. M. epaxialis and M. rectus cervicis are cut posteriorly.

The main muscles involved in the prey capture event are shown in figure 4, and are briefly described below. The M. depressor mandibulae consists of two distinct parts: M. depressor mandibulae posterior, and M. depressor mandibulae anterior. The M. depressor mandibulae posterior originates from the dorsal fasciae of the epaxialis musculature, and its fibres run ventrally to insert on the articular. The larger portion of the depressor system, the M. depressor mandibulae anterior, originates mainly from the os squamosum of the skull, runs ventrally and inserts slightly anterior to the insertion site of the M. depressor mandibulae posterior on the articular. The adductor system consists of two parts, the M. adductor mandibulae externus and the M. adductor mandibulae internus. The M. adductor mandibulae externus lies just anterior to the M. depressor mandibulae posterior. It originates from the anterior squamosum, runs ventrally and inserts on the coronoid part of the lower jaw. The M. adductor mandibulae internus has a rather complex arrangement including further subdivisions. The M. adductor mandibulae internus complex originates from the fasciae of the epaxialis musculature and more anteriorly from the parietale of the skull roof. Its fibres run ventrally to insert slightly anterior to the insertion site of the M. adductor mandibulae externus on the lower jaw. The M. depressor mandibulae complex inserts posterior and the M. adductor complex anterior to the jaw joint. The M. geniohyoideus originates medially on the dentary bone, close to the symphysis, and runs posteriorly to insert on the fasciae of the M. rectus cervicis. The M. rectus cervicis directly originates from the M. rectus abdominis (hypaxialis musculature) and runs anteriorly to insert on the proximal parts of hypobranchials 1 and 2 and on the median basibranchial part.

### 3.2. Kinematics

**Figure 5.**
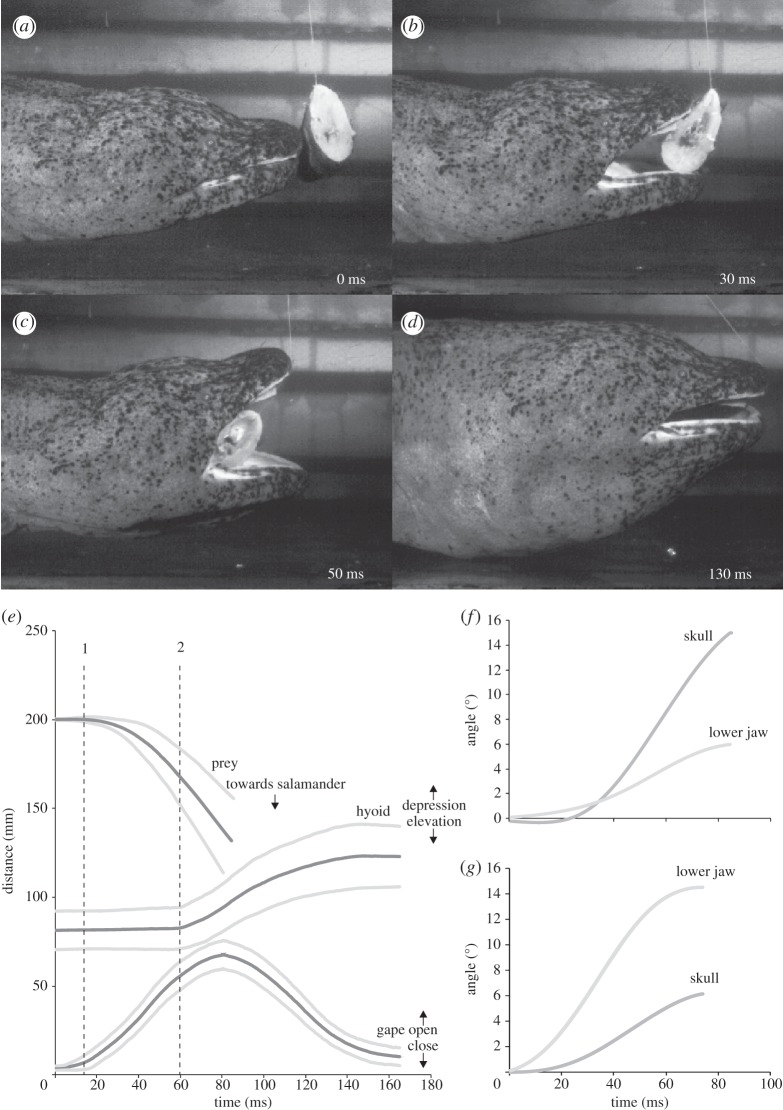
Frame shots (*a*–*d*) and mean kinematic profiles (*e*) of prey strike and angular jaw displacement (*f*,*g*) in *A. davidianus*. Note that hyoid depression starts shortly before the prey passes the mouth corners (*c*) and reaches its maximum as the mouth is closed (*d*). The kinematic profile shows means (±s.d.) of movements of gape, hyobranchium and prey. The first vertical dotted line (1) indicates the start of prey movement to the salamander mouth and the second (2) indicates the start of hyobranchial depression. The amount of angular rotation of the skull and lower jaw varied strongly between suction strikes, as shown in two extreme cases (*f*, skull rotation dominates; *g*, lower jaw rotation dominates), but the sum of both rotations resulted in more stereotypical mouth opening profiles (*e*).

Once detected, prey was slowly approached and then sucked in by a very fast and powerful suction strike. The strike started with mouth opening, caused by both dorsal skull rotation and ventral lower jaw rotation, followed by hyoid depression (figure 5).

Significant differences were found between individuals on 11 variables that best described the whole feeding event (MANOVA Wilks’ lambda *F* = 9.226; *p* ≤ 0.001). The subsequent series of ANOVAs revealed differences regarding the following distance and time variables: maximum gape distance (*F* = 26.9; *p* < 0.001), maximum hyoid depression (*F* = 23.8; *p* < 0.001), duration of mouth opening (*F* = 44.1; *p* < 0.001), duration of gape cycle (*F* = 17.7; *p* < 0.001) and delay of hyoid depression (*F* = 23.4; *p* < 0.001). No significant differences were detected between duration of mouth closing (*F* = 6.0; *p* = 0.008), duration of hyoid depression (*F* = 2.9; *p* = 0.071) and between all four velocity variables: velocity of mouth opening (*F* = 0.4; *p* = 0.705), velocity of mouth closing (*F* = 0.4; *p* = 0.7), velocity of hyoid depression (*F* = 1.8; *p* = 0.188) and velocity induced to the prey (*F* = 3.5; *p* = 0.048). A subsequently performed post hoc test (Tukey's honestly significant difference) further revealed that the smaller individual differed significantly (significance level, *p* ≤ 0.0046) from both other individuals in terms of maximum gape distance, duration of mouth opening, duration of gape cycle, delay of hyoid depression and maximum hyoid depression. By contrast, individuals 1 and 2 showed no significant differences concerning any of the variables tested. In other words, the difference between individuals detected by the MANOVA and the subsequent series of ANOVAs was mainly based on the differences in the prey strike between the smaller individual compared with the two larger ones.

Accordingly, the two larger individuals on the one hand, and the smaller one, on the other hand, have to be treated separately in the following descriptive kinematics. In the two larger animals, a mean maximum gape distance of 62.8 mm ± 8.53 (mean ± s.d.) was reached after 70.1 ± 7.3 ms, with a mean velocity of 0.91 ± 0.18 m s^−1^, immediately thereafter mouth closing started until the jaw plates met—or if prey was not completely engulfed and enclosed the prey item (figure 5*a–e*). The mouth closing movement was slightly slower than mouth opening, and lasted 84.6 ± 20.3 ms with a mean velocity of 0.78 ± 0.2 m s^−1^.

The duration of the whole gape cycle took 154.7 ± 22.4 ms and its averaged kinematical profile described a bell-shaped curve (figure 5*e*). The amount of angular rotation of the skull and lower jaw, and therefore their contribution to the mouth opening distance, varied strongly between suction strikes (figure 5*f,g*). The sum of both rotations, however, formed more stereotypical gape cycle profiles (figure 5*e*), resulting in linear maximum jaw displacement velocities of 1.3 ± 0.18 m s^−1^. Hyoid depression started with an average delay of 50.9 ± 9.64 ms after the start of mouth opening, and reached a maximum ventral deflection of 42.3 ± 9.1 mm at 85 ± 21.9 ms, with a mean velocity of 0.54 ± 0.21 m s^−1^. The suction strike induced a mean velocity to the prey of 0.95 ± 0.3 m s^−1^. Hyoid adduction started several seconds later and was very slow.

The movement of the prey towards the salamander's mouth started 15.5 ± 8.4 ms after initial mouth opening and was sucked completely into the mouth within 57.2 ± 9.5 ms. The suction mechanism of the two larger animals induced maximum prey accelerations towards the salamander of 40–50 m s^−2^, resulting in maximum prey speeds of 1.4 ± 0.5 m s^−1^. The total engulfed water volume, calculated for one adult specimen, was over 1.2 l.

The kinematical profile of the prey strike of the smaller salamander (62 cm total length) was very similar to that of the two larger ones (114 and 118 cm total length) described earlier, but due to the smaller size, maximum gape distance (40.5 ± 4.6 mm), duration of mouth opening (43.5 ± 4.1), duration of gape cycle (101.8 ± 20.3), delay of hyoid depression (25.7 ± 4.8) and maximum hyoid depression (26.3 ± 2.7) were significantly smaller. On the other hand, the two temporal variables, duration of mouth closing and duration of hyoid depression, as well as all four velocity variables, showed no significant differences to the two larger individuals.

All three animals, however, showed a highly significant correlation between the delay of hyoid depression (delay relative to start of mouth opening) and duration of mouth opening (*r*_22_ = 0.772; *p* < 0.001).

### 3.3. Hydrodynamics

Our CFD models calculated the three-dimensional unsteady flow in and around the salamander's mouth during four suction strikes. Note that the simulated flows were only the result of the upper and lower jaw movements: no ventral depression of the skin out of the plane between the lower jaw rami by action of the hyobranchium was included. Because such hyobranchium depression starts at 51 ± 9.6 ms after mouth opening, the model will only give a realistic image during the initial phase (i.e. time < 51 ms).

**Figure 6.**
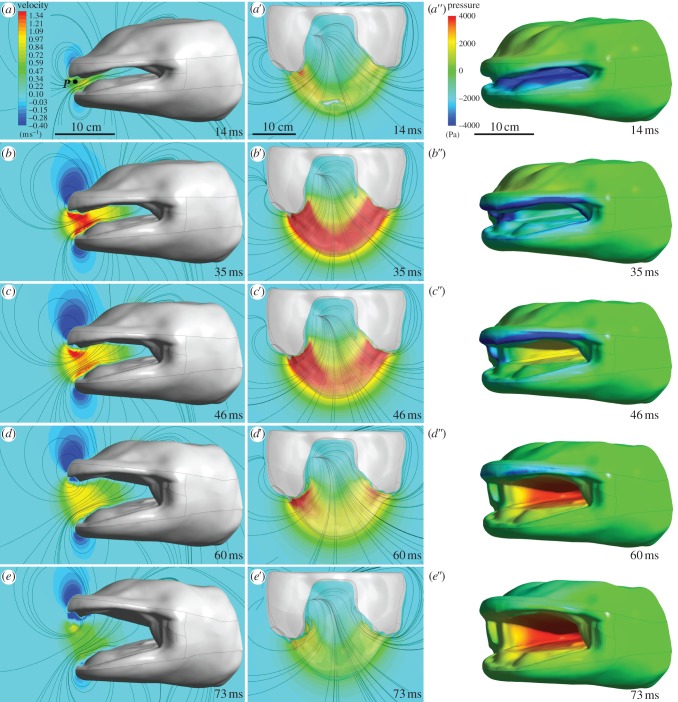
Flow patterns on the mid-sagittal (*a*–*e*) and corresponding midfrontal (*a*′–*e*′) plane and pressures on the surfaces of the salamander (*a*″–*e*″) for a mouth opening sequence calculated by CFD. Anterior-to-posterior flow velocities are shown in (*a*–*e*) so that negative values mean a posterior-to-anterior flow. Point *P* indicates a central, fixed position between the jaw tips where flow velocities are monitored (figure 7*a*). Absolute flow velocities are shown in (*a*′–*e*′). Streamlines (black lines) illustrate the instantaneous flow directions. The salamander's surface in (*a*′–*e*′) is made transparent to visualize intra-oral flow velocities. (*a*″–*e*″) Note the high negative pressure values on the internal oropharyngeal surface in the early mouth opening phase, which become positive 44 ± 8 ms after the start of mouth opening (defined as time = 0 ms).

**Figure 7.**
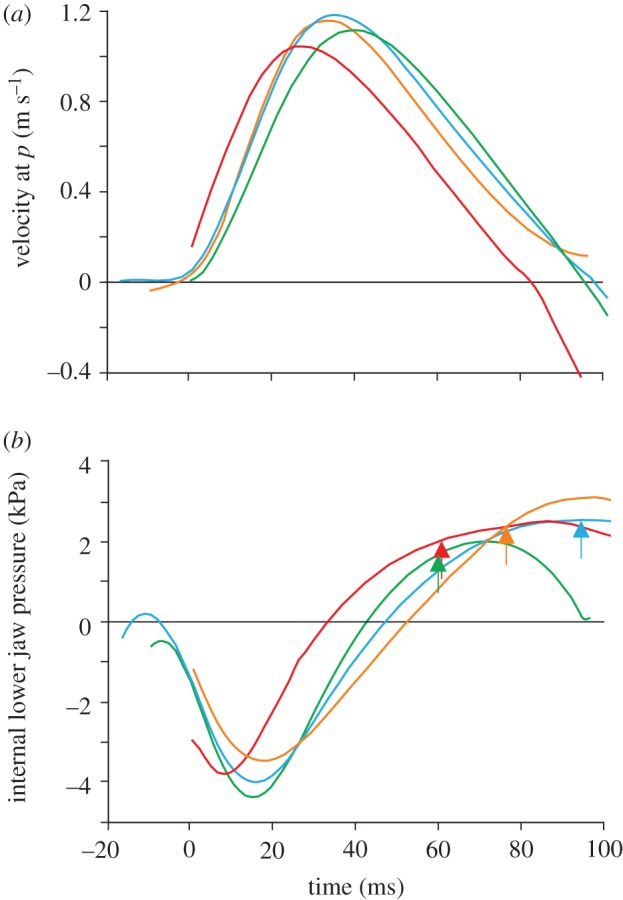
(*a*) Anterior-to-posterior flow velocity at point *P* (indicated in figure 6*a*) for four feeding sequences (coloured lines). (*b*) The corresponding surface-weighed mean pressure on the intra-oral face of the lower jaw. In (*b*), the approximate onset times of hyobranchial movement are indicated as coloured arrows.

Anterior-to-posterior flow velocities increased quickly after mouth opening started (figures 6*a–e* and 7*a*) and reached peak velocities after 31 ± 7 ms (figure 6*b*). We calculated peak flow velocities in the sagittal plane of 1.34 ± 0.07 m s^−1^ central in the mouth aperture, which slowly decreased towards the end of the mouth opening phase (figures 6*a–e* and 5*a*). Negative intra-oral flow velocities (i.e. posterior-to-anterior flow) were detected only close to the external surfaces of upper and lower jaws at the final instants of the jaw expansion. Water flows into the mouth from the sides at equal speed compared with from directly in front of the mouth, as shown by the absolute flow velocity plots in the midfrontal plane (figure 6*a*′–*e*′).

Instantaneous pressure acting on the oral surfaces during mouth opening was also quantified (figure 6*a*″*–e*″). Only 11 ± 6 ms after mouth opening started, intra-oral pressure dropped below −4.0 ± 0.3 kPa (figure 6*a*″), but soon increased again after reaching this negative peak. The pressure on the intra-oral surface between the left and right lower jaw bars, for example, became positive at 44 ± 8 ms, and reached its positive peak 84 ± 10 ms after the onset of mouth opening (figure 7*b*). Pressure changes on the external surfaces of the salamander during the simulated motion were negligible (figure 6*a*″–*e*″).

## 4. Discussion

Suction feeding evolved early in vertebrate history and is still widespread among aquatic predators: it is found in fishes [1–3,11,19–22], amphibians [6,7,9,12–18,25,38,39] turtles [4,5,23] and mammals [8,10,24]. Despite dramatic morphological and physiological differences among these groups, some general movement patterns to create rapid intra-oral pressure drop seem to be similar: the mouth is opened and hyobranchial depression creates oropharyngeal volume expansion that, due to the principle of continuity, results in a flow into the mouth. It is broadly accepted that such similarities are mainly the result of biophysical constraints to create a suction flow into the mouth rather than that of homologies. This becomes more evident if we consider that turtles and mammals, for example, independently evolved aquatic forms with suction feeding systems [8,40]. In contrast to these analogies, many features of the feeding mechanism of amphibians are ancestral characteristics that are retained from their sarcopterygian fish ancestors [41]. Most larval salamanders maintain the basic morphological configuration of the fish feeding mechanism with well-developed hyobranchium and associated muscles, as well as a large number of functional similarities in motor patterns and kinematics [41]. Even transformed (postmetamorphic) salamanders show a large number of ancestral features though their morphology has changed considerably [41,42].

The transition from larval to postmetamorphic salamanders includes, among other things, reduction and modification of the viscerocranium and the loss of gills and gill slits, and therefore a shift from a unidirectional to a bidirectional flow system when suction feeding [12–18]. The shift from a unidirectional to a bidirectional flow system (with all the associated morphological and functional changes) was shown to result in a drop in aquatic feeding performance in some salamander groups [14,16,17]. On the other hand, Miller & Larsen [39] reported high-performance aquatic prey capture by suction feeding in other species that—even after metamorphosis—retain a fully aquatic lifestyle. These species do have a modified (from their larval condition) hyobranchial system that is secondarily mineralized, with large hyobranchial elements compared with closely related terrestrial species [6]. This is consistent with previous assumptions that the salamander feeding system fundamentally relies on hyobranchial form and function [6,14,41], and a large, robust hyobranchium is advantageous for rapid hyoid depression as the main contributor to suction generation.

Our anatomical reconstruction showed that *A. davidianus* has a remarkably large, flattened skull and a broad snout. The lower jaw articulates with the quadrate far posteriorly at the level of the first cervical vertebra (figure 3), which highlights the dominant appearance of the lower jaw within the cranial system. The wide space between the lower jaws is covered ventrally by the hyobranchium. Interestingly, the hyobranchial skeleton is poorly ossified and mainly cartilaginous. This was surprising because we expected a stiff, bony hyobranchial skeleton, as it was generally assumed to be a prerequisite for most suction feeding metamorphosed salamanders where fast movements of the hyobranchium are the main contributor to suction generation [6,14,41].

In *A. davidianus*, a large fraction of the total cranial and post-cranial musculature can be recruited to power jaw opening. Ventral rotation of the lower jaw can be powered by contraction of the well-developed pair of mandibular depressor muscles, and might further be supported by contraction of the geniohyoideus muscle (figure 4). Additionally, the rectus cervicis muscle can have an indirect role in lower jaw depression, as its fasciae might offer stable insertion sites for the geniohyoideus muscle. However, because it is a direct extension of the rectus abdominis musculature (hypaxialis musculature) and inserts on the hyobranchial apparatus, its main function is probably posteroventral hyoid rotation. Dorsal skull rotation, the second contributor to jaw opening, is empowered by contraction of the massively developed epaxialis musculature that runs dorsally along the whole body and attaches to the occiput of the skull.

The motion analyses showed that during a suction strike in *A. davidianus*, the prey starts to move towards the salamander a few milliseconds after the beginning of mouth opening, and disappears into the mouth near the instant when the mouth reaches its maximum opening. Hyobranchial depression starts much later, only shortly before the prey passes the mouth corners. These results confirm the findings described for *Cryptobranchus alleganiensis* [26], suggesting that the considerable delay in hyobranchial depression is a general pattern for cryptobranchid salamanders. Because of the considerable prey motion during the phase in which only upper and lower jaw motion was observed, our kinematic data suggest that the rapid separation of the large and broad upper and lower jaw surfaces is solely responsible for the acceleration of the prey into the mouth. To test this hypothesis, we quantified the hydrodynamics resulting from the observed upper and lower jaw motion by CFD.

The CFD simulations showed that water between upper and lower jaw tips is accelerated into the mouth shortly after mouth opening started, reaching backwards velocities easily exceeding 1 m s^−1^. These flow velocity magnitudes are comparable to the values measured for high-performance unidirectional suction feeders [43–45] that, in contrast to our model, use considerable ventral depression of the hyoid elements much earlier in the expansive phase of suction. The values calculated from our CFD model are very similar to prey velocities measured on the original high-speed films. This validates the CFD model, and proves that high accelerations are induced to the prey by rapid jaw displacement, before motion in the hyobranchial region out of the plane of the lower jaw could be measured.

The positive intra-oral pressures at the time of onset of the balloon-like expansion of the ventral skin (figure 7*b*) strongly suggest that the water pushes these surfaces into expansion. Our model showed that pressure on the intra-oral surface between the left and right lower jaw bars becomes positive relatively early (44 ± 8 ms after the onset of mouth opening), which precedes the onset of ventral depression in the hyobranchial region (51 ± 9.6 ms). This means that at this instant, hyobranchial motion is assisted by decelerating water that pushes the oropharyngeal surfaces into expansion. In other words, the late expansion observed in the hyobranchial region is probably driven in part by a decreasing momentum of the sucked water when impacting these surfaces.

If the depression in the hyobranchial region out of the lower jaw plane were significantly empowered by contraction of the hypaxialis musculature, then a second peak of negative intra-oral pressure would be formed from 51 ms after mouth opening. This is highly unlikely since a double-peaked intra-oral pressure profile has never been observed in the numerous studies that measured pressures by implanted transducers in a wide variety of suction feeding vertebrates [3,10,14,21,22,25,41,46,47]. In addition, it is doubtful whether a functional advantage exists in an intermittent acceleration of a prey, as this would result in slower prey capture times compared with a single, continuous prey acceleration. Consequently, this reasoning gives indirect support that the expansions observed late in the suction phase (figure 5) are not only driven by hyobranchial retractor muscles (as observed in other suction feeding vertebrates), but to a large degree by the momentum of the water flow.

However, during the first stage of mouth opening by *A. davidianus*, the broad intra-mandibular coverage of the hyobranchium can play an important role in preventing the inward bending of the skin between left and right lower jaws, when the upper and lower jaw ‘plates’ separate to cause sub-ambient suction pressures of below −4 kPa. Without any skeletal support, intra-mandibular tissues (intra-mandibular muscles and skin) would probably be stretched towards the upper jaw, which will prevent further suction from being produced. Our videos show a very limited amount of dorsal motion of the intra-mandibular tissues with respect to the lower jaw (figure 5*b*; just anterior of the mouth).

**Figure 8.**
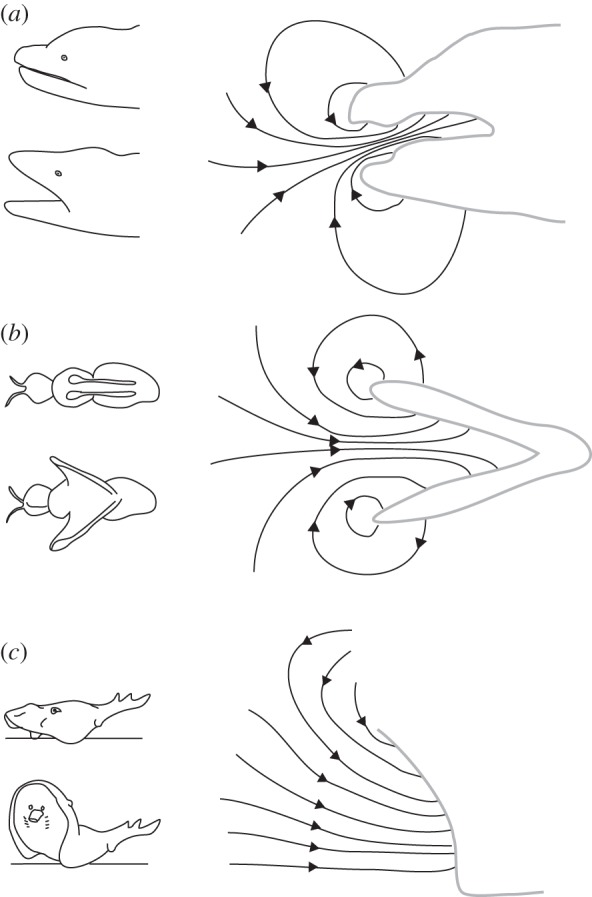
Examples of animals that generate suction through a quick separation of jointed surfaces: (*a*) the Chinese giant salamander, (*b*) insects using a ‘clap-and-fling’ of their wings during flight (adapted from [48–50]) and (*c*) rays lifting the rostrum from the substrate to draw water under the body to assist benthic feeding (adapted from [51,52]).

Generating suction by separation of two parallel surfaces in close apposition is not unique to the giant salamanders, as physical analogues can be found elsewhere in nature (figure 8). The ‘clap-and-fling’ of the wings of insects [48] relies on a similar fluid mechanical principle to generate additional lift during flight. During the fling, the dorsally clapped wings (acting essentially as rigid plates) will pronate about their trailing edge, creating a growing gap as the leading edges pull apart [49,50]. During this phase, a flow of air is ‘sucked’ downwards in between the separating wings and is accompanied by a body-lifting suction reaction force on the insect (owing to negative pressures) on the dorsally oriented wing surfaces. A second example shows that surface separation must not necessarily act between two body parts: benthic stingrays generate a strong, sustained suction flow by rapidly moving their flat rostrums upwards from the substrate to manipulate and capture prey [51,52] (figure 8). These examples show that suction can be generated effectively in other ways than the classical example of a radial expansion of a fluid-filled cavity as observed in suction feeding fishes [2].

We hypothesize that the function of the hyobranchial system, as described here for *A. davidianus*, has shifted during evolution. In primitive actinopterygian and sarcopterygian fishes, the hyoid apparatus plays a dominant role in producing suction pressure and is also involved in a biomechanical pathway to depress the mandible [53,54]. In *A. davidianus*, we found that it supports the medial surface in between the depressing mandible to function together as one of the two separating ‘plates’ that cause the acceleration of water and prey into the mouth. Afterwards, ventral motion of the hyobranchium serves to continue the flow of water into the buccopharyngeal cavity. The observation of a relative timing of hyobranchium depression showing a remarkably high stereotypy with respect to mouth opening in *A. davidianus* is in accordance with such a mechanism. Instead of a predominantly hyoid-powered suction mechanism as in fish, *A. davidianus* thus makes use of a jaw-powered suction system. Given the overall morphological similarity with *Cryptobranchus alleganiensis*, which also has a relatively small amount of hyobranchial ossification in a rather larval-like hyobranchium, and clear kinematic similarity during prey capture [26,38,55], a similar function of the feeding system can be expected for this species. The observed pattern is thus probably a general characteristic of giant salamanders (Cryptobranchidae).

Elwood & Cundall [55] described the ability for asymmetrical movements of jaws and hyobranchium during suction strikes in *C. alleganiensis*. In our study, food was always offered in front of the animals, and no asymmetrical movements were observed. However, from the morphological data obtained (more specifically, the cartilaginous mandibular symphysis and mainly cartilaginous hyobranchium with narrow symphyses), we deduce that *A. davidianus* might be capable, to a certain degree, of asymmetrical jaw and hyobranchial movements. Elwood & Cundall [55] used different prey types in their study and observed a high variability in suction movements. We fed only dead whole or halved fishes (roaches and trouts) and the salamanders showed symmetrical suction movements.

Because giant salamanders are one of the lineages retaining the most ancestral features among living tetrapods [27–30], the current analysis of their feeding biomechanics might shed new light on the feeding biology of early tetrapods [31,56]. Large, broad and dorsoventrally flattened skulls were characteristic for many Late Devonian tetrapod lineages, as well as for later branches [28]. Just like our model species, these early tetrapods were aquatic predators and their lifestyle was comparable to today's giant salamanders. Although some derived cranial features are present in giant salamanders compared with early tetrapods, these are outweighed by the striking overall morphological similarities. Consequently, it is not unlikely that rapid jaw displacement also played a central role in suction generation of early tetrapods.

More specifically, our data indicate that broad-skulled aquatic predators with large lower jaws can release the hyobranchium from its primitive suction-powering function during prey capture by using rapid jaw displacement to produce suction. In turn, in some branches, this may have allowed a further modification of the hyobranchial systems into a hyolingual system that can be used for terrestrial feeding purposes without losing performance in aquatic prey capture. Consequently, jaw-powered suction feeding might have been a potential key innovation in the transition to a terrestrial feeding lifestyle by allowing further development of a muscular, movable tongue. Furthermore, jaw-based suction feeding could also explain the large, broad and flattened ‘spade-shaped’ heads typically found in early amphibians [28] as a result of adaptive evolution.

In conclusion, our data suggest that *A. davidianus* uses a modified mechanism to generate suction compared with other lower vertebrates. Rapid displacement of the broad upper and lower jaws creates a quick drop in intra-oral pressure and an accelerating flow of water into the mouth. The inertia of the inflowing parcel of water assists a relatively late depression of the floor of the mouth by movement of the broad and cartilaginous hyobranchial apparatus. The latter movement helps to maintain the flow of water within the oral cavity, rather than generating the power stroke for prey capture. We hypothesize that such a biomechanical shift in the way that suction is powered (from hyobranchial- to jaw-powered suction) can be a key innovation in the fish–tetrapod transition: by releasing biomechanical constraints on the hyobranchial system, this may have opened the path for further modifications (e.g. the evolution of a fleshy tongue) without losing performance as aquatic predators.
